# Survival benefit of chemotherapy in metastatic colorectal cancer: a meta-analysis of randomized controlled trials

**DOI:** 10.1054/bjoc.1999.1254

**Published:** 2000-06

**Authors:** D J Jonker, J A Maroun, W Kocha

**Affiliations:** Ottawa Regional Cancer Centre and London Regional Cancer Centre, 790 Commissioners Road East, London, Ontario N6A 4L6, Canada

**Keywords:** advanced colorectal cancer, meta-analysis, phase III studies, antineoplastics, survival analysis, quality of life

## Abstract

To estimate the magnitude of benefit of chemotherapy in prolonging survival for patients with metastatic colorectal cancer, a meta-analysis of randomized controlled trial was performed. A systematic search was performed to identify randomized trials comparing chemotherapy with observation or supportive care alone. Trials were assessed for quality of reporting, publication bias and heterogeneity. Relative risks for outcomes from published data were pooled using a random-effects model. Seven trials with 614 patients were included. All trials used fluoropyrimidine-based chemotherapy, through a variety of routes and schedules, including intravenous, intra-portal and hepatic arterial infusion. Compared with the ‘no-chemotherapy’ arm, chemotherapy significantly reduced 1-year mortality (risk ratio 0.69; 95% confidence interval (CI) 0.60–0.81, *P*< 0.00001). The mortality at 2 years was not significantly different (risk ratio 0.93; 95% CI 0.87–1.00, *P* = 0.053). Between-trial comparisons demonstrated benefit with a variety of routes and schedules. Chemotherapy significantly prolongs 1-year survival for patients with metastatic colorectal cancer, and should be offered to those with good performance status. © 2000 Cancer Research Campaign


*British Journal of Cancer* (2000) **82**(11), 1789–1794
© 2000 Cancer Research Campaign
DOI: 10.1054/ bjoc.1999.1254, available online at http://www.idealibrary.com on IDEAL®

### Survival benefit of chemotherapy in metastatic colorectal cancer: a meta-analysis of randomized controlled trials

**DJ Jonker, JA Maroun and W Kocha**

Ottawa Regional Cancer Centre and London Regional Cancer Centre, 790 Commissioners Road East, London, Ontario N6A 4L6, Canada

**Summary** To estimate the magnitude of benefit of chemotherapy in prolonging survival for patients with metastatic colorectal cancer, a meta-analysis of randomized controlled trial was performed. A systematic search was performed to identify randomized trials comparing chemotherapy with observation or supportive care alone. Trials were assessed for quality of reporting, publication bias and heterogeneity. Relative risks for outcomes from published data were pooled using a random-effects model. Seven trials with 614 patients were included. All trials used fluoropyrimidine-based chemotherapy, through a variety of routes and schedules, including intravenous, intra-portal and hepatic arterial infusion. Compared with the ‘no-chemotherapy’ arm, chemotherapy significantly reduced 1-year mortality (risk ratio 0.69; 95% confidence interval (CI) 0.60–0.81, $P < 0.00001$). The mortality at 2 years was not significantly different (risk ratio 0.93; 95% CI 0.87–1.00, $P = 0.053$). Between-trial comparisons demonstrated benefit with a variety of routes and schedules. Chemotherapy significantly prolongs 1-year survival for patients with metastatic colorectal cancer, and should be offered to those with good performance status. © 2000 Cancer Research Campaign

**Keywords**: advanced colorectal cancer; meta-analysis; phase III studies; antineoplastics; survival analysis; quality of life

Colorectal cancer is the fourth leading malignancy, and the second leading cause of cancer death (National Cancer Institute of Canada, 1997). Metastases are present in 15% of patients at the time of diagnosis, and will develop in another 25% at some point in time. Except in a small minority of surgically resected cases (Foster and Berman, 1977), metastatic disease is uniformly fatal, with a median survival of 11 months in patients selected for clinical trials (Advanced Colorectal Cancer Meta-analysis Project, 1992). Chemotherapy, particularly fluoropyrimidines are used extensively for the treatment of colorectal cancer, though some physicians are skeptical regarding its benefits given the poor response rates reported, with pooled estimates of 11–23% (Advanced Colorectal Cancer Meta-analysis Project, 1992; Meta-analysis Group in Cancer, 1998).

Trials involving an observation or supportive care arm in advanced malignancies are difficult to perform, suffering from poor accrual, dropout and crossover. Therefore, any such available data are valuable, as they are not easily obtained.

Existing trials comparing chemotherapy with supportive care are not widely known, given their small size. A leading oncology text only references one study on this topic (Hine and Dykes, 1984; Cohen et al, 1997). This results in uncertainty for both patient and physician contemplating systemic therapy.

To estimate the magnitude of survival benefit of chemotherapy, a meta-analysis of randomized trials comparing chemotherapy with either observation or supportive care alone for metastatic colorectal cancer was conducted.

*Received 10 May 1999*
*Revised 8 October 1999*
*Accepted 17 November 1999*

*Correspondence to:* DJ Jonker

### METHODS

### Literature search

To identify randomized controlled trials of chemotherapy in metastatic colorectal cancer patients, a systematic search of the MEDLINE database from 1976 to 1998, the CANCERLIT database from 1983 to 1998, and the CURRENT CONTENTS database from 1997 was conducted using the following medical subject heading terms: colonic neoplasms, human, random allocation, research design, palliative care, and quality of life. In addition, the following text words were used in combination: random, colon, rectum, rectal, colorectal, cancer, carcinoma, adenocarcinoma, advanced, metastatic, metastases, metastasis, supportive, observation, delayed and control. The reference lists of retrieved articles were reviewed and further relevant studies were obtained. A hand search of recently published journals was performed, as well as a review of abstracts from proceedings of recent international meetings.

### Inclusion criteria

In order to be included in the pooled analysis, trials must have involved patients with stage IV or unresectable advanced colorectal cancer. Patients must have been randomized to either initial chemotherapy or initial management without chemotherapy, described as either initial observation or supportive care alone.

### Assessment for publication bias

A funnel plot was generated to assess for possible publication bias (Lau et al, 1997). Trial size was plotted against the risk ratio for

**Table 1** Randomized clinical trials comparing chemotherapy versus initial observation in metastatic colorectal cancer

| Trial | No. of patients | Treatment | Randomization method described | Intention to treat | Median survival in months (*P*-value) | 1-year survival | 2-year survival |
|---|---|---|---|---|---|---|---|
| Hine et al | 26 | 5-FU/meCCNU i.v. | No | Yes | 16 (= 1.0) | 73% | 23% |
| | 26 | Observation ± 5-FU | | | 16 | 73% | 23% |
| Rougier et al | 81 | HAI FUDR | No | Yes | 15 (NR) | 63% | 23% |
| | 82 | Observation or 5-FU | | | 11 | 44% | 13% |
| NGTATG | 92 | MTX/LV/5-FU i.v. | Yes | Yes | 15 (= 0.13) | 55% | 15% |
| | 90 | Observation/delayed CT | | | 9 | 38% | 12% |
| Scheithauer et al | 24 | CDDP/LV/5-FU i.v. | Yes | No[a] | 11 (= 0.006) | 42% | 12% |
| | 12 | BSC | | | 5 | 8% | 0% |
| Hafstrom et al | 32 | HA occlusion/intraportal 5-FU | No | No[b] | 16 (NR) | 62% | 31% |
| | 28 | Observation ± 5-FU/LV | | | 8 | 36% | 18% |
| Allen-Mersh et al | 51 | HAI FUDR | No | Yes | 13 (= 0.3) | 51% | 18% |
| | 49 | BSC | | | 7 | 35% | 16% |
| Glimelius et al | 11 | 5-FU/LV i.v. | No | Yes | 12 (= 0.1) | 45% | NR |
| | 10 | Observation ± 5-FU/LV | | | 6 | 30% | NR |

[a]Excluded 2 control patients and 2 treatment patients not accepting assignment after randomization; no details given. [b]Excluded 2 control patients and 2 treatment patients not meeting inclusion criteria after randomization. i.v., intravenous; HAI, hepatic-arterial infusion; 5-FU, 5-fluorouracil; FUDR, fluorouridine-deoxyribose; MTX, methotrexate; LV, leucovorin; BSC, best supportive care; NR, not reported.

each trial, to determine if small, negative studies were probably left unpublished.

### Quality assessment

All randomized controlled trials comparing an active treatment arm using antineoplastics with a control arm using initial observation or supportive care were retrieved. Trials were assessed independently for quality of trial reporting by three reviewers using components of the Jadad instrument (Jadad et al, 1996), and for analysis performed on an intent-to-treat basis. Studies were excluded if both survival curves and survival data were not available. When disagreement existed, it was resolved through conference.

### Data extraction

Using published survival curves or directly published data, the 1-year and 2-year mortality was determined for the chemotherapy and supportive care arms. Data regarding response rates, quality of life and toxicity were summarized quantitatively and qualitatively. Median, 1-year and 2-year survivals in each trial were also summarized.

### Primary end point

In the primary analysis, the 1-year overall survival of patients treated with chemotherapy versus supportive care was assessed. For each study, a relative risk estimate was calculated as the mortality in the chemotherapy arm divided by the mortality in the control arm. The individual relative risk estimates were pooled and a 95% confidence interval (CI) was provided using a random-effects model (DerSimonian and Laird, 1986) computed with Meta-Analyst 0.998 (c 1998, J Lau). Survival was considered statistically significant if the 95% CI for the relative risk did not encompass 1. Heterogeneity between the trials was determined by calculating the Q statistic using a $\chi^2$ test (Fleiss, 1981).

### Secondary end points

Secondary outcome measures included 2-year overall survival, quality of life and a sensitivity analysis of 1-year overall survival. The sensitivity analysis was conducted by combining subgroups of trials based on patient and treatment differences between trials. Subgroups compared include carcinoembryonic antigen (CEA)-positive only versus measurable or evaluable disease, localized hepatic metastases versus diffuse metastatic spread, and route of administration.

### RESULTS

### Trials selected

From the initial search, 860 abstracts were identified and reviewed online. From these, 12 articles were retrieved of which six met the inclusion criteria. A further three trials were identified by reference and hand search of journals.

Seven of these nine randomized controlled trials were used in the meta-analysis. They had a total of 614 patients comparing chemotherapy to an initial observation arm in patients with metastatic colorectal cancer (Hine and Dykes, 1984; Nordic Gastrointestinal Tumour Adjuvant Therapy Group (NGTATG), 1992; Rougier et al, 1992; Scheithauer et al, 1993; Allen-Mersh et al, 1994; Hafstrom et al, 1994; Glimelius et al, 1995). These are listed in Table 1, along with a brief description of treatment schedules. One trial by Beretta et al (1994) published only in abstract form was excluded due to insufficient data reported for survival analysis. Cunningham et al (1998) was excluded as it involved second-line therapy. Chemotherapy was fluoropyrimidine based all of the trials (5-fluorouracil (5-Fu) in five and FUDR in two), and was administered by a variety of routes (intravenous in four, hepatic intra-arterial in two, and portal venous in one). Statistically there was no significant heterogeneity in 1-year survival between trials (Q statistic = 3.53, $P > 0.1$). A funnel plot (not shown) did not identify any evidence of publication bias, though this cannot be entirely excluded.

 *© 2000 Cancer Research Campaign*

**Table 2** Patient characteristics

| Trial | Accrual period | Treatment arm | No. of patients | M/F | Median age | Colon/ rectum | Performance status |
|---|---|---|---|---|---|---|---|
| Hine et al | ??–1983 | 5-FU/meCCNU | 26 | 18/8 | 61 | NR | KPS 100% |
| | | Observation ± 5-FU | 26 | 15/11 | 59 | NR | KPS 100% |
| Rougier et al | 1985–1988 | HAI FUDR | 81 | 47/34 | 59 | 52/29 | 94% ECOG 0–1 |
| | | Observation or 5-FU | 82 | 44/38 | 61 | 58/24 | 92% ECOG 0–1 |
| NGTATG | 1985–1990 | MTX/LV/5-FU | 92 | 63/37 | 60 | 62/38 | 84% KPS 100% |
| | | Observation/delayed CT | 90 | 51/49 | 60 | 79/21 | 84% KPS 100% |
| Scheithauer et al | 1988–1989 | CDDP/LV/5-FU | 24 | 10/14 | 63 | 14/10 | 67% ECOG 0–1 |
| | | BSC | 12 | 7/5 | 69 | 8/4 | 67% ECOG 0–1 |
| Hafstrom et al | 1984–1992 | HA occlusion/intraportal 5-FU | 32 | 16/12 | 57 | 17/11 | NR |
| | | Observation ± 5-FU/LV | 28 | 10/16 | 62 | 20/6 | NR |
| Allen-Mersh et al | 1988–1993 | HAI FUDR | 51 | 34/17 | 55 | NR | KPS 90% |
| | | BSC | 49 | 29/20 | 59 | NR | KPS 90% |
| Glimelius et al | 1991–1992 | 5-FU/LV | 11 | NR | 65 | 6/5 | KPS 78% |
| | | Observation ± 5-FU/LV | 10 | NR | 61 | 6/4 | KPS 80% |

NR, not reported; M/F, male/female; KPS, Karnofsky performance status; HAI, hepatic-arterial infusion; 5-FU, 5-fluorouracil; FUDR, fluorouridine-deoxyribose; MTX, methotrexate; LV, leucovorin; BSC, best supportive care.

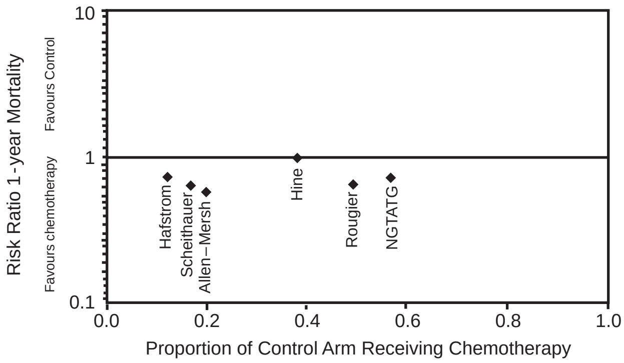

**Figure 1** A plot illustrating the effect of chemotherapy use in control arm patients (contamination) on risk ratio of 1-year mortality

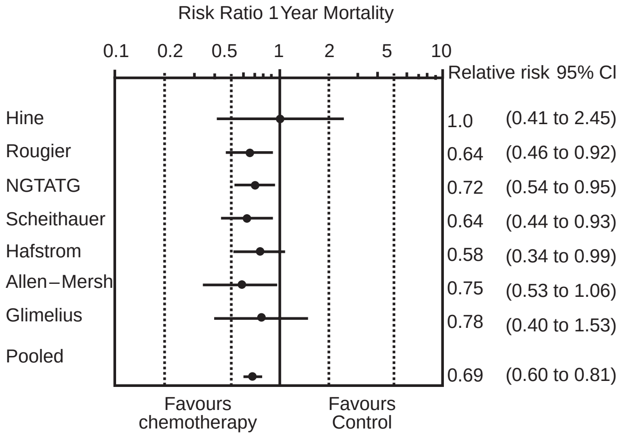

**Figure 2** A plot of risk ratio of 1-year mortality with 95% confidence intervals (CI) for each trial and the pooled estimate

### Quality assessment

None of the available trials used placebo controls and there was no attempt to blind either patient or physician. In choosing the hard end point of mortality, the risk of bias by the assessing physician is reduced. Trial quality is reported in Table 1. Two trials violated intent-to-treat analysis with inappropriate exclusion of a total of four control patients and four treatment patients.

### Patient characteristics

All trials, except Hine and Dyke (1984), involved patients with measurable or evaluable disease. This trial included patients with rising CEA without other evidence of metastases. Patient characteristics (where published) are recorded in Table 2. The median age was 60 years. Fifty-seven per cent were male. Sixty-eight per cent were colon primaries and 32% rectal. Patients were well balanced between groups for sex, age and performance status. Most patients had excellent performance status (Karnofsky performance status 80–100) at entry into the trials.

### Chemotherapy in the supportive care arm

Many trials allowed in their design for delayed or discretionary use of chemotherapy for patients randomized to the observation or supportive care arm. Chemotherapy was received by 12–57% of control patients, as reported in Figure 1. These patients were all analysed on an intention-to-treat basis. Subsequent chemotherapy use by patients in the control arm did not to reduce the benefit of use of initial chemotherapy ($P = 0.682$).

### Pooled analysis

Figure 2 shows the relative risk of 1-year mortality in each trial with individual and pooled estimates. Chemotherapy resulted in a significantly lower risk of mortality at 1 year compared to no chemotherapy (risk ratio 0.69; 95% CI 0.60–0.81, $P < 0.00001$). The odds ratio for 1-year mortality was 0.48 (95% CI 0.34–0.66, $P = 0.00001$). In patients with a 42% 1-year mortality risk, which was the rate for the pooled sample from these seven trials of generally good performance status patients, the number needed-to-treat (NNT) with chemotherapy to result in one additional patient alive at 1 year is 5.4 patients (95% CI 3.9–9.2) with a risk difference of 1-year mortality of –0.1835 (95% CI –0.2586 to –0.1084, $P < 0.00001$).

Figure 3 shows the relative risk of two-year mortality in each trial with individual and pooled estimates. There was no significant

*© 2000 Cancer Research Campaign* 

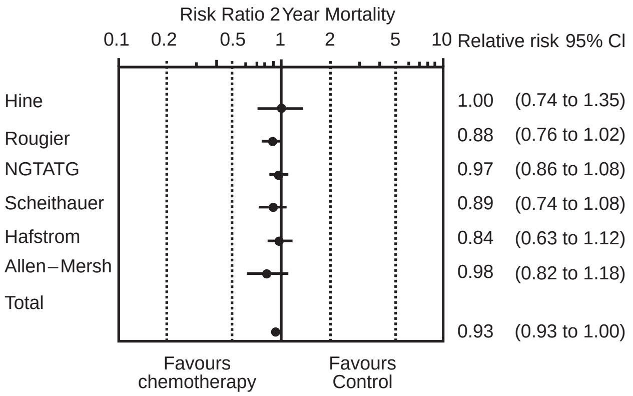

**Figure 3** A plot of risk ratio of 2-year mortality with 95% confidence intervals (CI) for each trial and the pooled estimate

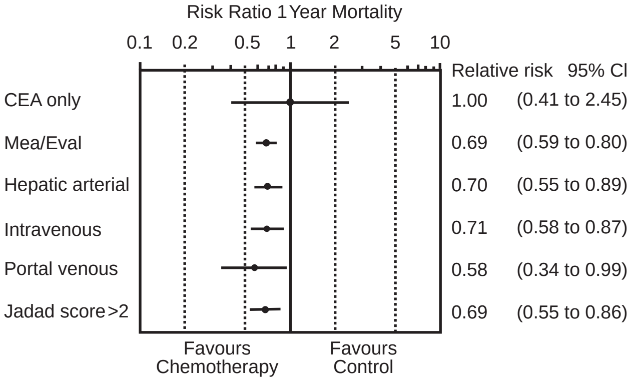

**Figure 4** A plot of risk ratio of 1-year mortality with the 95% confidence intervals (CI) for various subgroups of trials based on characteristics of patients at entry (measurable/evaluable versus CEA-positive only), route of administration of chemotherapy, and the quality of trial reporting

**Table 3** Quality of life measures

| Trial | Measure | Initial chemotherapy | Control | *P*-value |
|---|---|---|---|---|
| NGTATG | Median symptom-free survival | 10 months | 2 months | < 0.001 |
| | Median progression-free survival | 8 months | 3 months | < 0.001 |
| Scheithauer et al | Preservation of QOL by FLIC score | 67% | 62% | > 0.5 |
| Allen-Mersh et al | Rotterdam checklist and HAD scale | – | – | > 0.5 |
| Glimelius et al | Median quality-adjusted survival | 9.6 months | 4.2 months | NR |

QOL, Quality of life; FLIC, Functional Living Index for Cancer; HAD, Hospital Anxiety and Depression; NR, Not reported.

difference, with a relative risk of 0.93 (95% CI 0.87–1.00, $P$ = 0.053). The risk difference was –0.0617 (95% CI –0.11 to –0.0035, $P$ = 0.038) and the odds ratio was 0.66 (95% CI 0.43–1.03, $P$ = 0.067).

### Sensitivity analysis for 1-year mortality results

Figure 4 shows estimates for risk ratio of 1-year mortality for patients treated with chemotherapy for various patient and treatment subgroups through between trial comparisons. All subgroups appeared to benefit from chemotherapy, except CEA-only positive patients, for whom there was insufficient power to exclude a benefit. Sensitivity analysis for 2-year overall survival found that only the subgroup with measurable or evaluable disease had a statistically significant improvement in survival, with a risk ratio for 2-year mortality of 0.93 (95% CI 0.86–1.00, $P$ = 0.047).

### Quality of life

Quality of life was evaluated in four trials by a variety of measures, including the FLIC (Scheithauer et al, 1993), the Rotterdam checklist and the Hospital Anxiety and Depression Scale (Allen-Mersh et al, 1994). In these studies, there was either maintenance of, or improvement in, the scores of the chemotherapy arm compared to the initial observation arm (Table 3).

### Toxicity

Toxicity was not quantitatively documented by many of the trials. Where documented, toxicity was generally mild to moderate. In Hafstrom et al (1984), there was unacceptable toxicity in the chemotherapy arm with two patient deaths related to incorrect dosages of chemotherapy used for a portal venous infusion, constituting major protocol violations. In Rougier et al (1992), hepatic arterial infusion was associated with a 25% incidence of sclerosing cholangitis by 1 year.

### DISCUSSION

Before interpreting the data, some caution is advised with meta-analysis of published data. Publication bias (the existence of missed unpublished negative studies) may result in an over-estimation of the true treatment effect, which would not occur with a single, large randomized study. The funnel plot did not suggest a publication bias, but this cannot be excluded. One trial published in abstract form, which included 88 elderly patients with colorectal cancer, was not included, as data was not available. This would represent 12% of the total sample had they been included. The abstract suggested a survival benefit, but there was inadequate information to include in the pooled analysis.

Caution must also be taken in interpretation of the subgroup analyses based on between-trial comparisons. They do not

*© 2000 Cancer Research Campaign*

adequately take into account differences between patients within trials. These analyses may be hypothesis generating, but many comparisons will be better assessed by individual patient data meta-analysis (for example the effect of performance status on treatment effect), or ideally randomized controlled trials (for example, whether HAI is superior to intravenous chemotherapy).

This meta-analysis of randomized controlled trials shows that chemotherapy results in a significant survival advantage compared with non-chemotherapy management. Of patients randomized to chemotherapy, there was an 18% absolute reduction in 1-year mortality, resulting in one additional patient alive for every 5.4 patients treated. This NNT would apply to a patient with a baseline 1-year mortality of 42%, which was the pooled 1-year mortality rate in these combined trials of generally good performance status patients. The benefit in terms of a median survival difference varies between populations treated, and ranged from 0 to 8 months difference (median 6 months). To determine the difference more precisely, an individual patient data meta-analysis would be required.

The benefit appears consistent for a variety of regimens and regardless of route of administration. The drugs used varied between trials, and many were inferior by today's standards. Although all included fluoropyrimidines, the basis of all conventional standard first-line chemotherapy regimens, these were combined with drugs such as cisplatin, methotrexate and methyl CCNU, drugs which are considered to be inferior, unnecessary, or associated with increased toxicity compared to 5-FU and leucovorin (LV) combinations.

Similarly, the route of administration did not appear to alter the survival benefit. For patients with localized unresectable liver metastases, both local therapies, such as portal venous infusion and hepatic arterial infusion, and systemic treatment appear to confer a survival benefit.

The patients in Hine and Dyke (1984) were CEA-positive without other evidence of measurable or evaluable disease, representing an earlier stage of disease than the other trials. The sample size was too small to detect a potentially clinically significant difference. No other trials have evaluated chemotherapy in this setting, making recommendations difficult. This trial does not support or counter the use of chemotherapy in this population, though many would feel uncomfortable withholding treatment in this subgroup. A randomized trial using conventional chemotherapy would be valuable in this population, though accrual would be difficult.

The results of this meta-analysis are further supported by Cunningham et al (1998) comparing second-line irinotecan to supportive care in this same population. This demonstrated a 36.2% 1-year survival in this irinotecan group versus 13.8% in the supportive-care group. This is a risk ratio of 0.74 for 1-year mortality. The survival benefit remained significant after adjustment for prognostic factors in a multivariate analysis ($P = 0.001$).

The trials evaluated suffer some methodological problems related to having a non-treatment arm. In particular, there was no blinding, risking bias in softer end points such as quality of life or response rates. The use of hard end points, such as mortality, will reduce this risk. The inappropriate exclusion of eight patients from analyses in these trials may have resulted in an over- or underestimation of the true treatment effect.

Patients included in these trials were of generally high performance status. One cannot necessarily generalize these results to patients in poorer general condition. It is interesting to note, however, that the difference in median survival was as significant in the trial by Scheithauer et al (1993), which included patients with poorer performance status, suggesting the survival benefit may not be limited to those with high performance status.

One question that remains unanswered is whether initial or delayed chemotherapy is superior in an asymptomatic patient. In some of these trials there was a high rate of delayed use of chemotherapy by patients in the control arm. The NGTATG (1992) trial in particular had chemotherapy use in 57% of the control patients, allowed as part of the study design. This trial suggests that immediate chemotherapy is superior to delayed chemotherapy. The answer is being addressed in ongoing trials, including an Australian study and an NCI Canada study (NCIC CO-10) which has closed prematurely due to poor accrual. The pooled results from these two studies may clarify this issue.

The fact that many patients in the control arms received chemotherapy as part of their supportive care would have weakened any observed difference between the treatment and observation arms. This crossover to anti-tumour therapy may have weakened any survival benefit observed, thus strengthening the results of this meta-analysis.

Despite the demonstrated benefit, the outcomes of patients treated with chemotherapy are still poor. Much work is needed, particularly in the arena of new drug development, in order to improve the outcome for patients with colorectal cancer.

### ACKNOWLEDGMENT

DJ Jonker was supported by the Ontario division of the Canadian Cancer Society through the Gordon E Richards Fellowship.

### REFERENCES

Advanced Colorectal Cancer Meta-analysis Project (1992) Modulation of fluorouracil by leucovorin in patients with advanced colorectal cancer: evidence in terms of response rate. *J Clin Oncol* **10**: 896–903

Allen-Mersh TG, Eatlam S, Fordy C, Abrams K and Houghton J (1994) Quality of life and survival with continuous hepatic-artery floxuridine infusion for colorectal liver metastases. *Lancet* **344**: 1255–1260

Beretta G, Bollina R, Labianca E, Arnoldi E, Luliri P and Matignoni G (1994) A controlled study of supportive care versus supportive care plus 5-fluorouracil/folinic acid for advanced metastatic gastrointestinal carcinomas in the elderly patient. *Proc Ann Meet Am Soc Clin Oncol* **13**: 221 (abstract 669)

Cohen AM, Minsky BD and Schilsky RL (1997) *The Principles and Practice of Clinical Oncology*, 5th edn, Devita V (ed), p. 1177. Lippincott-Raven: Philadelphia

Cunningham D, Pyrhonen S, James RD, Punt CJ, Hickish TF, Heikkila R, Johannesen TB, Starkhammar H, Topham CA, Awad L, Jacques C and Herait P (1998) Randomized trial of irinotecan plus supportive care versus supportive care alone after fluorouracil failure for patients with metastatic colorectal cancer. *Lancet* **352**: 1413–1418

DerSimonian R and Laird NM (1986) Meta-analysis in clinical trials. *Controlled Clin Trials* **7**: 177–188

Fleiss JL (1981) *Statistical Methods for Rates and Proportions*, 2nd edn, pp. 161–165. J Wiley: New York

Foster HJH and Berman MM (1977) Solid liver tumours. In: *Major Problems in Clinical Surgery*, Vol 22, Ebert PA (ed), pp. 209–234. WB Saunders: Philadelphia, PA

Glimelius B, Hoffman K, Graf W, Haglund U, Nyren O, Pahlman L and Sjoden PO (1995) Cost-effectiveness of palliative chemotherapy in advanced gastrointestinal cancer. *Ann Oncol* **6**: 269–274

Hafstrom L, Engaras B, Holmberg S, Gustavsson B, Jonsson PE, Lindner P, Naredi P and Tidebrant G (1994) Treatment of liver metastases from colorectal cancer with hepatic arterial occlusion, intraportal 5-fluorouracil infusion, and allopurinol. *Cancer* **74**: 2749–2756

Hine KR and Dykes PW (1984) Prospective randomized trial of early cytotoxic therapy for recurrent colorectal carcinoma detected by serum CEA. *Gut* **25**: 682–688

© 2000 Cancer Research Campaign

Jadad AR, Moore RA, Carroll D, Jenkinson C, Reynolds DJM, Gavaghan DJ and McQuay HJ (1996) Assessing the quality of reports of randomized clinical trials: is blinding necessary? *Controlled Clin Trial* **17**: 1–12

Lau J, Ioannidis JPA and Schmid CH (1997) Quantitative synthesis in systematic reviews. *Ann Int Med* **127**: 820–826

Meta-analysis Group in Cancer (1998) Efficacy of intravenous continuous infusion of fluorouracil compared with bolus administration in advanced colorectal cancer. *J Clin Oncol* **16**: 301–308

National Cancer Institute of Canada (1997) *Canadian Cancer Statistics*, 10th edn. NCIC: Toronto, Canada.

Nordic Gastrointestinal Tumour Adjuvant Therapy Group (1992) Expectancy or primary chemotherapy in patients with advanced asymptomatic colorectal cancer: a randomized trial. *J Clin Oncol* **10**: 904–911

Rougier P, Laplanche A, Huguier M, Hay JM, Ollivier JM, Escat J, Salmon R, Julien M, Roullet Audy JC, Gallot D, Gouzi JL, Pailler JL, Elisa D, Lacaine F, Roos S, Rotman N, Luboinski M and Lasser P (1992) Hepatic arterial infusion of floxuridine in patients with liver metastases from colorectal carcinoma: long term results of a prospective randomized trial. *J Clin Oncol* **10**: 1112–1118

Scheithauer W, Rosen H, Kornek GV, Sebesta C and Depisch D (1993) Randomized comparison of combination chemotherapy plus supportive care with supportive care alone in patients with metastatic colorectal cancer. *Br Med J* **306**: 752–755

*© 2000 Cancer Research Campaign*
